# Structural Characterization and Antioxidant Activities of Polysaccharides Extracted from the Pulp of *Elaeagnus angustifolia* L.

**DOI:** 10.3390/ijms150711446

**Published:** 2014-06-26

**Authors:** Qingqing Chen, Juncheng Chen, Hongtao Du, Qi Li, Jun Chen, Gechao Zhang, Hong Liu, Junru Wang

**Affiliations:** 1 College of Science, Shihezi University, Shihezi 832003, XinJiang, China; E-Mail: chenqingqing1027@gmail.com; 2 College of Science, Northwest A&F University, Yangling 712100, Shaanxi, China; E-Mails: chenjc@gmail.com (J.C.); duht@nwsuaf.edu.cn (H.D.); zhanggc@gmail.com (G.Z.); 3 Department of Biology, Yangling Hi-Tech Middle School, Yangling 712100, Shaanxi, China; E-Mails: liqi@gmail.com (Q.L.); chenj@gmail.com (J.C.)

**Keywords:** *Elaeagnus angustifolia* L., polysaccharides, isolation, structural characterization, antioxidant activity

## Abstract

In this study, two polysaccharides (*Elaeagnus angustifolia* L. polysaccharide-1 (PEA-1) and PEA-2) were prepared from *Elaeagnus angustifolia* L. Then, the preliminary structure and antioxidant activities of all the samples were investigated. The results showed that the average molecular weights for PEA-1 and PEA-2 were 9113 and 5020 Da, respectively. And, PEA-1 was mainly composed of rhamnose, xylose, mannose, glucose, and galactose, respectively. The components of PEA-2 were rhamnose, mannose, glucose, and galactose, respectively. Moreover, the Antioxidant assays demonstrated that PEA-1 possessed of strong free radicals scavenging activity and hydroxyl radicals scavenging activities, suggesting that PEA-1 could potentially be used as natural antioxidant.

## 1. Introduction

*Elaeagnus angustifolia* L., namely shazao in China, contains multiple chemical constituents, including proteins, flavonoids, amino acids, polysaccharides, and inorganic elements, *etc*. [1]. And, it has a variety of medicinal uses. Such as it is employed in the treatment of nausea, vomiting, jaundice, asthma, and flatulence [2,3,4]. The pulp of *Elaeagnus angustifolia* L. have been used to treat amoebic dysentery. Antibacterial agents, such as epigallocatechin from the bark of E. glabra, were identified [5]. The flavonoid of *Elaeagnus angustifolia* L. showed significant antinociceptive activity and muscle relaxant activity [6,7].

The polysaccharides, as an important component of *Elaeagnus angustifolia* L., have anti-radiation activity, immunoregulatory activity, and antioxidant activity [8,9]. Our group reported that the crude polysaccharide could reinforce immune function of experimental mice [10]

Based on the above information, the aim of this work is to prepare pure polysaccharides of *Elaeagnus angustifolia* L. fruits, and investigate the preliminary characterization, antioxidants, *in vitro*.

## 2. Results and Discussion

### 2.1. Isolation and Purification of Polysaccharides

**Figure 1:**
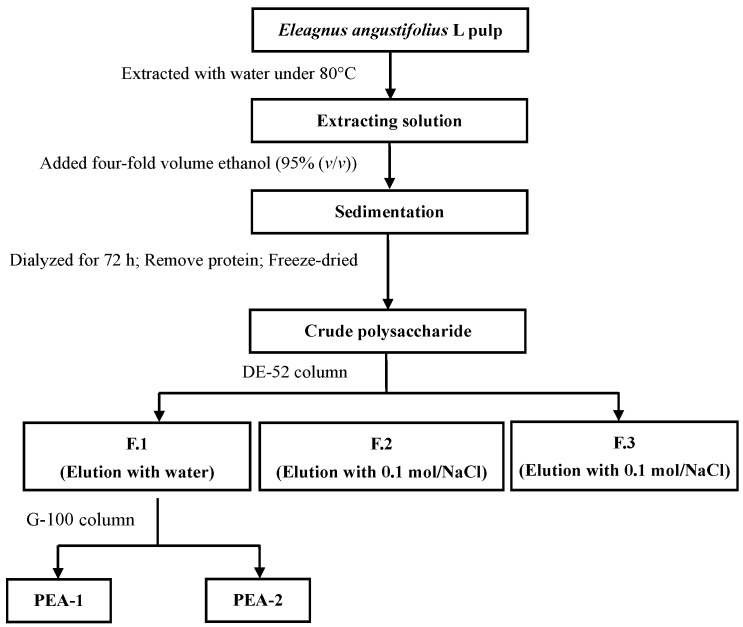
Extraction and isolation scheme.

The crude polysaccharide was extracted from *Elaeagnus angustifolia* L. pulp. The crude polysaccharide was isolated three fractions using a deae cellulose 52 (DE-52) column. The neutral polysaccharide fraction, eluted with water, was further fractionated using a G-100 column, and a major extracellular polysaccharides PEA-1 and PEA-2 was obtained (Figure 1).

### 2.2. Preliminary Characterization of Elaeagnus angustifolia L. Polysaccharide (PEA)

#### 2.2.1. Molecular Weight of PEA

**Figure 2:**
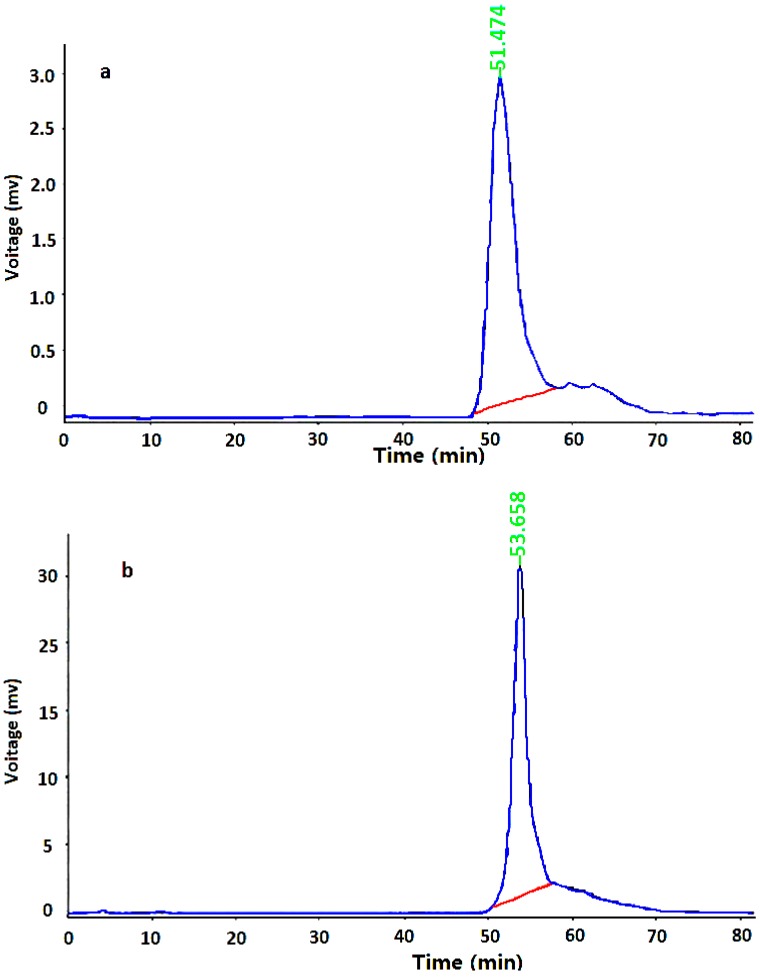
HPGPC chromatograms of (**a**) PEA-1and (**b**) PEA-2 of molecular weigths.

HPLC system was used to determine the homogeneity and molecular weight of PEA-1 and PEA-2. As shown in Figure 2, the HPLC profile of each purified fraction had a single and symmetrical narrow peak, which indicated that they were homogeneous polysaccharides. In addition, the average molecular weights of PEA-1 and PEA-2 were estimated to be 9113 and 5020 Da, respectively.

#### 2.2.2. Ultraviolet (UV) and Fourier Translation Infrared (FT-IR) Spectrometric Characterization of PEA

**Figure 3:**
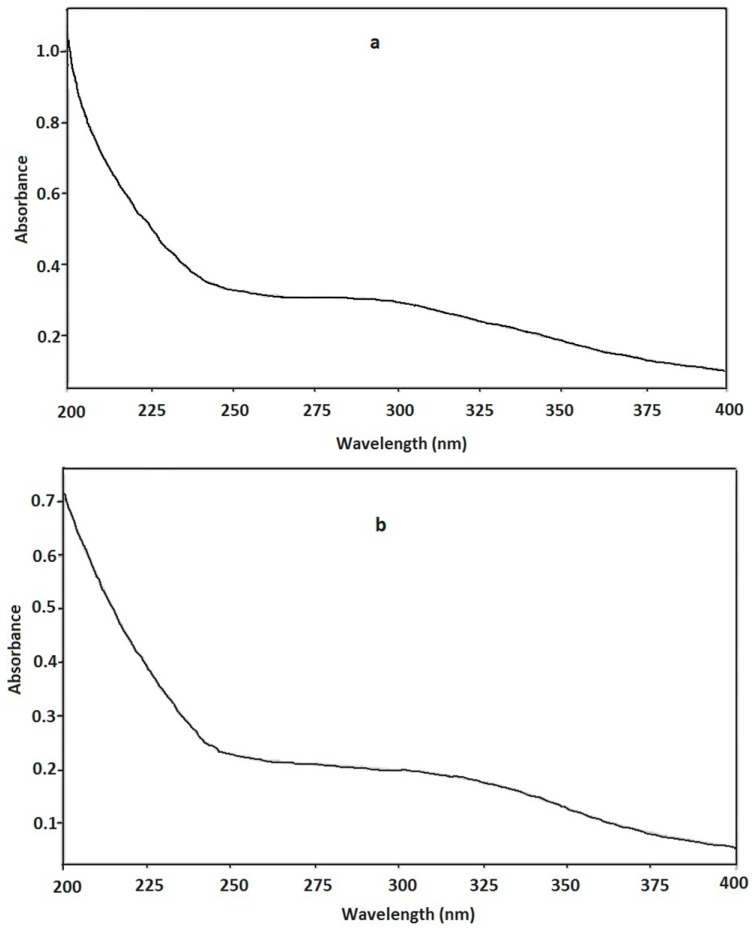
The UV spectra of (**a**) PEA-1 and (**b**) PEA-2.

The UV spectra of PEA are shown in Figure 3. No significant absorption at 260–280 nm was observed in the UV spectrum of PEA-1 and PEA-2, indicating the presence of impossible protein.

**Figure 4:**
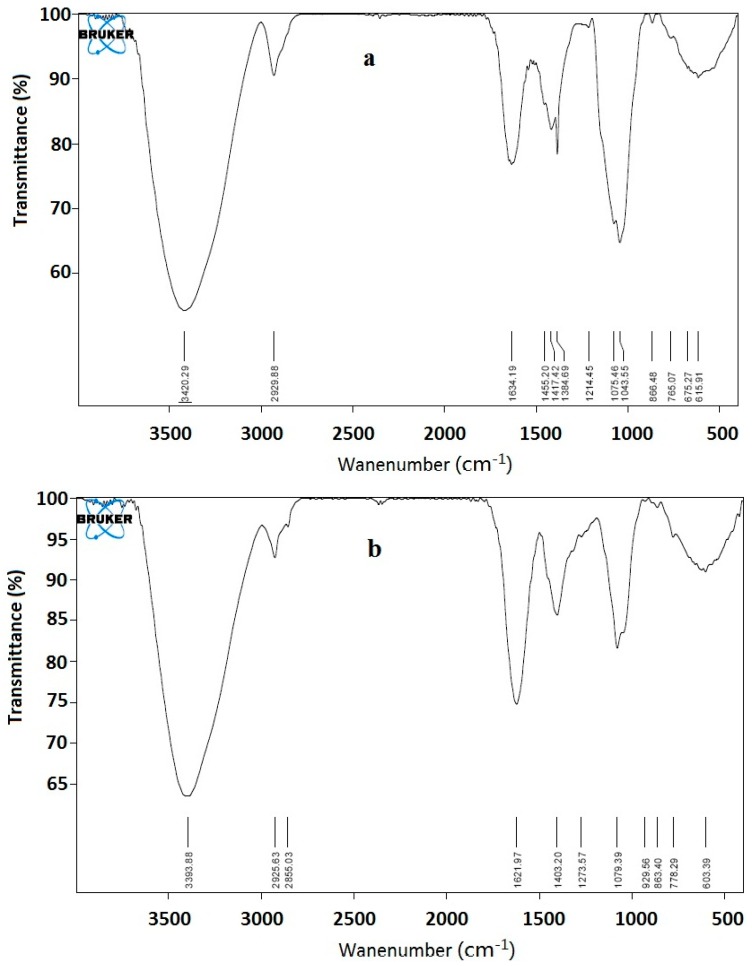
IR spectrum of (**a**) PEA-1and (**b**) PEA-2.

An FT-IR spectroscopy is used to investigate the vibrations of molecules and polar bonds between the different atoms. It is possible to analyze the structures of polysaccharides, such as monosaccharide types, glucosidic bonds, and functional groups, using an FT-IR spectroscopy. PEA-1 and PEA-2 were characterized by FT-IR spectroscopy (Figure 4). A strong and broad absorption peak at 3400 cm^−1^ for O–H stretching vibrations, a peak at 2930 cm^−1^ for C–H stretching vibrations, and a strong extensive absorption in the region of 900–1200 cm^−1^ for coupled C–O and C–C stretching and C–OH bending vibrations were observed in crude PEA-1 and PEA-2, indicating the characteristic absorptions of polysaccharides.

#### 2.2.3. Sugar Compositions of PEA-1 and PEA-2

The monosaccharide composition of PEA-1 and PEA-2 were determined by gas chromatograph-mass spectrometer (GC-MS) and compared with the monosaccharide standards. The result showed that PEA-1 was mainly composed of rhamnose, xylose, mannose, glucose, and galactose, respectively, and the component of PEA-2 were rhamnose, mannose, glucose, and galactoserespectively (Table 1).

**Table 1:** Components of monosaccharide of polysaccharides.

Sample	Monosaccharide Composition (Relatively Mass %)					Polysaccharide Content (%)
	Rha.	Xyl.	Man.	Glu.	Gal.	
PEA-1	37.1	1.6	6.3	35.4	19.6	98.8
PEA-2	12.4	ND ^a^	7.1	42.4	38.1	97.3

ND ^a^: not detected.

### 2.3. Antioxidant Activity

#### 2.3.1. Effect of Scavenging 1,1-Diphenyl-2-picryl-hydrazyl (DPPH) Radicals

**Figure 5:**
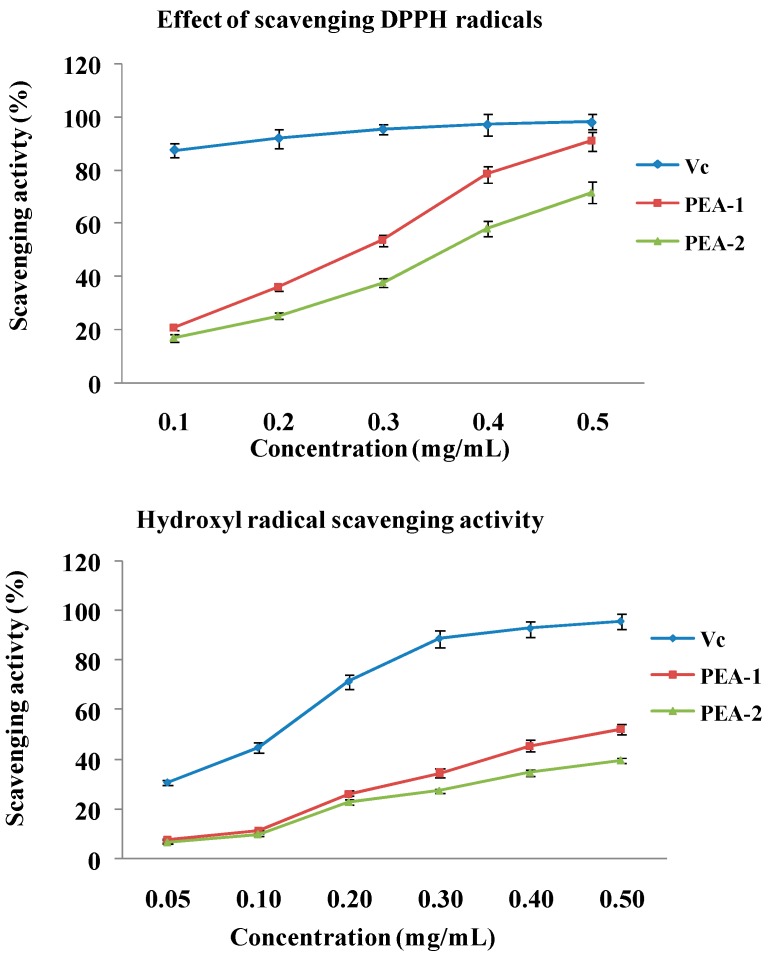
DPPH radical scavenging activities and hydroxyl radical scavenging activities of PEA-1, PEA-2 and Vitamin C (Vc). Each value is the Mean ± standard deviation (SD) of triplicate measurements.

1,1-Diphenyl-2-picryl-hydrazyl (DPPH) assay is based on the reduction of DPPH radicals in the presence of a hydrogen donating antioxidant, resulting in the formation of a stable non-radical form DPPH-H and color fades ups. As shown in Figure 5, PEA-1 and PEA-2 showed obvious scavenging effect on DPPH radical in a dose dependent manner. At the concentrations ranged from 0.1 to 0.5 mg/mL, the scavenging ability on DPPH radical of PEA-1 was much higher than PEA-2. At the concentration of 0.5 mg/mL, PEA-1 possessed strong free radical scavenging effects of DPPH radicals (scavenging activity 88.1%). The IC_50_ values of scavenging DPPH radicals were 0.27 and 0.38 mg/mL for PEA-1 and PEA-2.

#### 2.3.2. Measurement of Hydroxyl Radical Scavenging Activity

The hydroxyl radical is considered to be mainly responsible for oxidative injury of biomolecules. As shown in Figure 5, all samples exhibited obvious scavenging activity on hydroxyl radical in a dose dependent pattern at all concentrations. PEA-1 had the stronger activity than PEA-2. At the 0.5 mg/mL, the scavenging activities of PEA-1and PEA-2 were 52.3% and 39.8%, respectively. The scavenging hydroxyl radical activity was attributed to various mechanisms, such as suppression against hydroxyl radical generation, decomposition of peroxides, prevention of continued hydrogen abstraction, and radical scavenging. The possibility of scavenging hydroxyl radical by polysaccharide, was that polysaccharide could combine with radical and terminate the radical chain reaction [11].

PEA-1 and PEA-2 have different structures and monosaccharide compositions, which lead to the antioxidant difference between PEA-1 and PEA-2. PEA-1 is mainly composed of rhamnose (37.1%), glucose (35.4%), and galactose (19.6%); the component of PEA-2 major are rhamnose (12.4%), glucose (42.4%), and galactose (38.1%). The major difference is the different content of rhamnose and galactose between PEA-1 and PEA-2. But, the absolute configuration of polysaccharide is difficult to confirm. So, the antioxidant difference between PEA-1 and PEA-2 is hard to explain clearly.

## 3. Experimental Section

### 3.1. Materials and Supplies

*Elaeagnus angustifolia* L. was purchased from local market (Shihezi, Xijiang, China). The pulp was separated manually from the fruit. Then powdered, and kept at room temperature for study.

Monosaccharides (d-glucose, l-rhamnose, d-xylose, l-arabinose, l-mannose, l-fucose, d-galactose) were purchased from Sigma-Aldrich Co. (Saint Louis, MO, USA). Trifluoroacetic acid, DEAE-Cellulose, Sephadex G-100, 1-diphenyl-2-picryl-hydrazyl, and other chemicals were obtained from New Sanli chemical reagents company (Xi’an, Shaanxi, China.).

### 3.2. Isolation and Purification of the Extracellular Polysaccharide

The powder of *Elaeagnus angustifolia* L. pulp was extracted with water at 80 °C. Then the extraction was filtered through cheesecloth. The liquids were concentrated under reduced pressure at 40 °C, and four-fold volume of 95% (*v*/*v*) ethanol was added. The resulting precipitate was recovered by using centrifugation at 3500 rpm for 10 min, and dialyzed in cellulose membrane (molecular weight cut-off 3500) against flowing distilled water for 72 h. The retained fraction was recovered, concentrated under reduced pressure, and freeze-dried. The solution of crude polysaccharide was mixed with Sevage reagents (chloroform-butanol 4:1, *v*/*v*), based on certain percentage under magnetic stirring for 20 min, to remove protein at the liquid-junction. Then, the crude polysaccharide was fractionated on DEAE-Cellulose 52 column (Beijing Huamaike Biotechnology Co., LTD, Beijing, China) elution with step-wise gradient of 0, 0.1 and 0.3 mol/L (NaCl solution). The total sugar content of the fractions was determined by the phenol-sulfuric acid method. Subfractions eluted with water, were pooled, dialyzed, and further purified on Sephadex G-100 column (Shanghai Haoran Biological Technology Co., LTD, Shanghai, China) with water as eluent. The major subfractions (PEA-1 and PEA-2) were pooled and freeze-dried.

### 3.3. Determination of Total Polysaccharide Yield

The polysaccharide content was measured by phenol-sulfuric method using d-glucose as a standard. The yield of crude polysaccharide was calculated as a percentage of the weight of the dry sample weight. The percentage total polysaccharide yield (%) is calculated as follows:

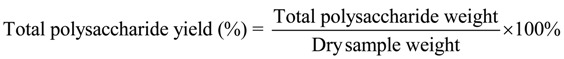
(1)


### 3.4. Preliminary Characterization of PEA-1 and PEA-2

#### 3.4.1. Determination of Molecular Weights

The molecular weight determination of PEA was performed using size-exclusion HPLC chromatography. All samples (10.0 mg) were dissolved in distilled water (1.0 mL), passed through a 0.45 µm filter, and applied to a gel-filtration chromatographic column. The column was maintained at a temperature of 25 °C and eluted with 0.1 M Na_2_SO_4_ solution in PBS buffer (0.01 M, pH 6.8) at a flow rate of 0.8 mL/min.

#### 3.4.2. UV and FT-IR Spectrometric Analysis

UV spectrum of PEA was recorded with a UV-2450 spectrophotometer (Shimadzu, Kyoto, Japan). FT-IR spectrum of PEA was recorded with a FT-IR spectrometer (Tensor 27, Baruker, Bremen, Germany) using the KBr disks method. Briefly, samples were dried at 35–44 °C in vacuum over P_2_O_5_ for 48 h, ground with potassium bromide (KBr) powder, and then pressed into pellets for FT-IR spectral measurement in the frequency range of 4000–400 cm^−1^.

#### 3.4.3. Analysis of Monosaccharide Composition

The compositional analysis of PEA-1 and PEA-2 were performed by the alditol acetate method [12], with minor modifications. Firstly, the polysaccharide was hydrolyzed by 2 M trifluoroacetic acid at 121 °C for 3.5 h, followed by reduction in distilled water with NaBH_4_ 1.5 h at room temperature. Secondly, hydrolysate was acetylated by acetic anhydride with pyridine as the catalyst at 100 °C for 1 h. Finally, the alditol acetate derivatives produced were separated by gas chromatography (GC) (7890A-G3440A, Agilent, Palo Alto, CA, USA).

### 3.5. Determination of Antioxidant Activities of PEA-1 and PEA-2 in Vitro

#### 3.5.1. Effect of Scavenging DPPH• Radicals

The scavenging activity on DPPH free radical (DPPH•) was measured as described previously [13,14,15], with slight modification. Briefly, 0.1 mM solution of DPPH• in methanol was prepared and 1.0 mL of this solution was added to 3.0 mL of the polysaccharides of various concentrations (0.1, 0.2, 0.3, 0.4, and 0.5 mg/mL) in water. The mixture was shaken and incubated at 25 °C for 30 min in the dark. Then the absorbance was measured at 517 nm. Vitamin C (Vc) was used as the positive control.

#### 3.5.2. Assay of Hydroxyl Radical (•OH) Scavenging Activity

The hydroxyl radical (•OH) scavenging activity was measured according to Fenton’s reaction [13,16,17]. The hydroxyl radical was generated in a mixture of 1.0 mL of 5 mM 1,10-phenanthroline, 1.0 mL of 0.05 M sodium phosphate buffer (pH 7.4), 0.5 mL of 7.5 mM FeSO_4_ and 0.5 mL of H_2_O_2_ (3%, *v*/*v*). After addition of 2.0 mL sample solution, the mixture was incubated at 37 °C for 1 h. The absorbance was measured at 510 nm. Deionized water and Vc were used as the blank and positive control, respectively.

### 3.6. Statistical analysis

All data were expressed as mean ± standard deviation (SD).

## 4. Conclusions

In the present study, the preliminary characterization, antioxidants *in vitro* of polysaccharides from *Elaeagnus angustifolia* L. were investigated. Preliminary structural analysis indicated that PEA-1 and PEA-2 were mainly composed of rhamnose, glucose and galactose *etc*., respectively, and the average molecular weights for PEA-1 and PEA-2 were 9113 and 5020 Da, respectively. Antioxidant activities *in vitro* demonstrated that PEA-1 and PEA-2 had moderate scavenging activity and lipid peroxidation inhibition effect. These results suggested that PEA-1 and PEA-2 had potent antioxidant activities.

In order to develop and use it effectively, we will work on the animal-testing, which be used to evaluate the food safety and application potential.
